# Biosynthesis and Degradation of Sulfur Modifications in tRNAs

**DOI:** 10.3390/ijms222111937

**Published:** 2021-11-03

**Authors:** Naoki Shigi

**Affiliations:** Cellular and Molecular Biotechnology Research Institute, National Institute of Advanced Industrial Science and Technology (AIST), 2-4-7 Aomi, Koto-ku, Tokyo 135-0064, Japan; naoki-shigi@aist.go.jp

**Keywords:** iron–sulfur cluster, mitochondria, post-transcriptional modification, sulfur, translation

## Abstract

Various sulfur-containing biomolecules include iron–sulfur clusters that act as cofactors for enzymes, sulfur-containing vitamins such as thiamin, and sulfur-modified nucleosides in RNA, in addition to methionine and cysteine in proteins. Sulfur-containing nucleosides are post-transcriptionally introduced into tRNA molecules, where they ensure precise codon recognition or stabilization of tRNA structure, thereby maintaining cellular proteome integrity. Modulating sulfur modification controls the translation efficiency of specific groups of genes, allowing organisms to adapt to specific environments. The biosynthesis of tRNA sulfur nucleosides involves elaborate ‘sulfur trafficking systems’ within cellular sulfur metabolism and ‘modification enzymes’ that incorporate sulfur atoms into tRNA. This review provides an up-to-date overview of advances in our knowledge of the mechanisms involved. It covers the functions, biosynthesis, and biodegradation of sulfur-containing nucleosides as well as the reaction mechanisms of biosynthetic enzymes catalyzed by the iron–sulfur clusters, and identification of enzymes involved in the de-modification of sulfur atoms of RNA. The mechanistic similarity of these opposite reactions is discussed. Mutations in genes related to these pathways can cause human diseases (e.g., cancer, diabetes, and mitochondrial diseases), emphasizing the importance of these pathways.

## 1. Introduction

**Figure 1:**
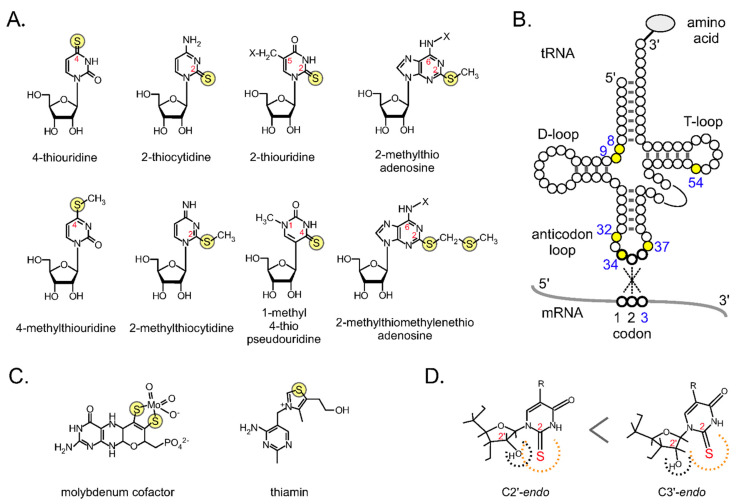
Sulfur-containing tRNA modifications: (**A**) Chemical structures of thiolated nucleosides. (**B**) Secondary structure of tRNA and the positions of thiolated nucleosides in tRNA. The codon triplet (codon positions 1, 2, and 3) base pairs with positions 36, 35, and 34, respectively, in the anticodon of the tRNA. (**C**) Examples of sulfur-containing cofactors. (**D**) Conformation of xm^5^s^2^U. Due to the steric hindrance of the 2-thio and 2′-OH groups, the C3′-*endo* form is preferred. This figure was modified from Figure 1 in [3].

Sulfur is one of the six major elements (i.e., hydrogen, oxygen, carbon, nitrogen, phosphorus, and sulfur) accounting for 99% of living organisms. Although cysteine and methionine are the most well-known sulfur-containing molecules in living organisms, there are various other sulfur-containing biomolecules, including iron–sulfur clusters, that act as cofactors for enzymes, sulfur-containing vitamins such as thiamin, and sulfur-modified bases of RNA (Figure 1). A variety of sulfur-containing secondary metabolites have also been identified [1]. Sulfur atoms can exist in many allotropes (more than 30 as single isotopes) and can stably form compounds with a wide range of oxidation numbers (from −2 to +6). This flexibility makes it possible for sulfur to exist in a variety of forms and perform diverse functions in living organisms.

These flexible chemical characteristics of sulfur have made experimental analysis difficult, but significant efforts have been made. The detection of sulfur compounds that reflect the actual forms in cells can be achieved by appropriately controlling the redox state, stabilizing the compounds by chemical modification, and applying mild detection conditions [2]. In addition, the use of an oxygen-free experimental apparatus has become widespread, especially for molecules that are susceptible to oxidation.

Through these innovations, we now know that for the biosynthesis of sulfur-modified bases of tRNA, organisms have evolved sophisticated systems to prevent secondary reactions and to allow only the desired reaction to proceed, which is essential for accurate protein synthesis [3]. For safe handling, highly reactive activated sulfur species are covalently attached transiently to carrier proteins. The same mechanism has been demonstrated for the biosynthesis of iron–sulfur clusters and sulfur-containing vitamins, which are also important for many life processes [4]. As our understanding of specific biosynthetic systems for sulfur compounds is deepening, common underlying principles are becoming clearer.

Abnormalities in sulfur modifications in tRNAs are a cause of serious diseases (cancer, diabetes, mitochondrial diseases, etc.) [5,6], and oxidative stress can cause diseases accompanied by abnormalities in sulfur-modified bases of tRNAs [7]. Fundamental studies have been conducted using model prokaryotic cells to human cells and purification of specific RNAs with liquid chromatography-mass spectrometry (LC-MS) characterization of modified nucleosides [8,9]. Next-generation sequencing, such as ribosome profiling and combining high-throughput sequencing with traditional biochemical methods using modification-specific antibodies or chemical derivatization, is now commonplace [10]. Preparation of recombinant proteins and RNA substrates has been used to quantitatively analyze enzymatic reactions in vitro, and mouse models lacking RNA-modifying enzymes as well as patient-derived cells are widely used in research on the mechanisms of development of diseases.

In this review, the functions and biosynthetic mechanisms of sulfur-modified bases are summarized, and the latest basic research on the reaction mechanisms of RNA sulfur-(de)modifying enzymes possessing iron–sulfur clusters is presented. For detailed structural aspects of RNA sulfurtransferases, please refer to an excellent review on the subject in [11].

## 2. Variations and Their Functions of Sulfur Modification of RNA

Proteins are the major functional components of living organisms, and, as enzymes, they catalyze the chemical reactions that support life. In protein synthesis, tRNA is the central molecule that assigns the codons of genes to the amino acids of proteins. There are more than 110 chemical modifications in tRNAs [12], among which sulfur-modified bases are essential for accurate codon recognition [3,13]. After the transcription of tRNA genes, precursor tRNAs undergo cleavage and chemical modification and mature into functional molecules. tRNAs have many modifications around their anticodons that are important for accurate codon recognition (Figure 1A,B). The 2-thiouridine (s^2^U) modification is found at position 34 (first letter of the anticodon) of Glu, Gln, and Lys tRNAs in almost all organisms (position 5 of the base is also modified and important). Steric hindrance of the bulky 2-thio group and the 2′-hydroxyl group stabilizes the C3′-*endo* form (Figure 1D) [14,15] and contributes to ‘wobble’ binding to the third codon [16,17,18,19]. Loss of this modification reduces the speed of translation and causes stress to cells as aggregates accumulate because nascent proteins fail to adopt their proper conformations [20]. More recently, in addition to codon content, the presence of hydrophilic motifs was suggested to contribute to aggregation [21]. The 2-methylthioadenosine (ms^2^A) modification is located at position 37 (next to the anticodon) in many organisms, and it is important for accurate codon reading by strengthening base pairing between the third letter of the U anticodon and the first letter of the A codon [22] (in humans, it is found in cytoplasmic tRNA-Lys, mitochondrial tRNA -Trp, -Phe, -Tyr, and -Ser [6,7]. A hyper-modified nucleoside, 2-methylthiomethylenethioadenosine (msms^2^A), was identified in *Escherichia coli* tRNAs [23].

In bacteria, many tRNAs have 4-thiouridine (s^4^U) at the root of the acceptor stem (positions 8 and 9) (Figure 1A,B). It has been reported that 4-thiouridine (s^4^U) absorbs ultraviolet (UV) light of ~340 nm and acts as a near-UV sensor by intramolecular cross-linking with C at position 13 to stall translation [24,25,26]. Recently, it was reported that s^4^U stabilizes the conformation of tRNA and prevents its degradation in the cell [27]. A 2-thiocytidine (s^2^C) modification at position 32 on the anticodon loop of tRNA also affects codon recognition [28]. Recently, methylation damage of *E. coli* tRNAs was reported to produce 2-methylthiocytidine (ms^2^C; [29]) and 4-methylthiouridine (ms^4^U; [30]) modifications (for details, see Section 4.2). In addition, some thermophilic bacteria have a 2-thiouridine modification on the T-loop (position 54) of almost all tRNAs, and the percentage of tRNAs that are thiolated increases with increasing environmental temperature [31,32,33]. Sulfurization stabilizes the core double-stranded structure of the elbow region of L-shaped tRNAs formed with a D-loop and T-loop. This interaction improves the thermal stability of the entire tRNA molecule [34], hence sulfurization at position 54 is essential for the growth of thermophilic bacteria at high temperatures [35]. The hyperthermophilic archaeon *Ignicoccus hospitalis* has a novel modified nucleoside 1-methyl-4-thiopseudouridine (m^1^s^4^Ψ) at position 54 [36]. The methyl and thiol groups in m^1^s^4^Ψ54 are spatially identical to m^5^s^2^U54 in the T-loop, meaning that two distinct thiolated nucleosides have evolved convergently to stabilize the L-shaped structure of whole tRNAs.

## 3. Biosynthesis Pathways of Sulfur Modification of RNA

Although the sulfur donors for RNA modifications in vivo are still largely unknown, in bacteria (*E. coli*, *Salmonella typhimurium*, *Bacillus subtilis*, etc.) the sulfur in thiouridines is derived from intracellular L-cysteine; the sulfur atom of L-cysteine is covalently bound to cysteine desulfurase, a pyridoxal-5′-phosphate (PLP)-dependent enzyme, on the catalytic cysteine residue, forming a persulfide (R-SSH)-activated sulfur species [35,37,38,39,40,41]. The enzyme-bound persulfide is then passed on to sulfur carrier proteins, and finally the desired sulfur-modified base is biosynthesized by sulfurtransferases (generally referred to as modifying enzymes) in each pathway [42].

Cysteine desulfurase is the primary enzyme that provides activated sulfur not only to sulfur-modified bases but also to biosynthetic systems such as iron–sulfur (Fe–S) clusters, thiamin, and molybdenum cofactors (Moco) [43,44,45,46]. Each pathway also shares downstream sulfur carrier proteins and activated sulfur intermediates, forming a so-called sulfur metabolic network in which each pathway is mutually influenced [47,48]. This intracellular biosynthetic network of sulfur compounds is a sophisticated system that safely handles highly reactive and toxic activated sulfur species, and it achieves an orderly flow of sulfur atoms thanks to the involvement of a large number of sulfur carrier proteins. Interestingly, it was recently reported that there are more persulfide species (from small molecules to protein-bound forms) in cells than previously expected [49,50], but their roles in the biosynthesis of sulfur-containing compounds await elucidation. In some archaeal cells, there are high concentrations (low mM range) of sulfides that are thought to be directly introduced into RNA by modification enzymes without any sulfur carrier proteins [51].

### 3.1. tRNA Uridine Sulfurtransferase Ncs6/TtuA

**Figure 2:**
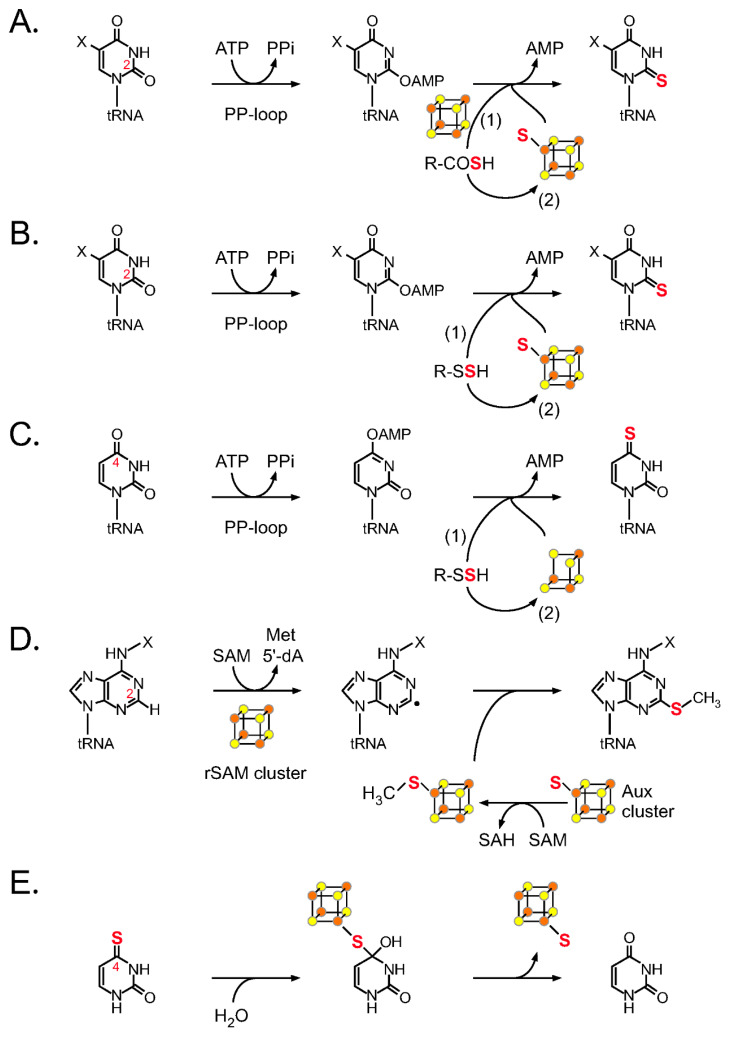
Reaction mechanisms of representative tRNA sulfurtransferases and a desulfidase: (**A**) Reaction mechanism of sulfurization of uridine C2 by TtuA/Ncs6. A protein-bound thiocarboxylate is introduced into the tRNA either (1) directly or (2) once sulfur is transferred to the iron–sulfur cluster. (**B**) Mechanism of sulfurization at uridine C2 by MnmA. The protein-bound persulfide acts as a sulfur donor. A protein-bound persulfide is introduced into the tRNA either (1) without the aid of the iron–sulfur cluster or (2) once sulfur is transferred to the iron–sulfur cluster. (**C**) Mechanism of sulfurization at uridine C4 by ThiI. The protein-bound persulfide acts as a sulfur donor. In general (1), a protein-bound persulfide is introduced into the tRNA without the aid of the iron–sulfur cluster. Unusually in MmThiI (2), a [3Fe–4S]-type cluster is required for sulfur transfer. (**D**) Mechanism of methyl sulfation of adenosine C2 by MiaB. Methyl sulfide formed on the Aux cluster is introduced into tRNA. SAM, S-adenosyl-L-methionine; SAH, S-adenosyl-L-homocysteine; 5′-dA, 5′-deoxyadenosyl radical. (**E**) Desulfidation reaction of 4-thiouracil catalyzed by TudS. A sulfur atom is captured on the [4Fe–4S] cluster to form the [4Fe–5S] state. This figure was modified from Figure 2 in [3].

**Figure 3:**
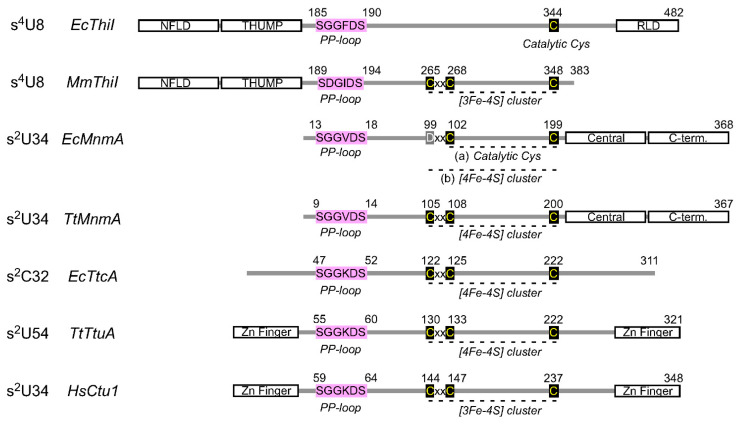
Domain organization of tRNA uridine/cytidine sulfurtransferases. The conserved residues in the active domain (center) and the accompanying RNA-binding domain are shown. Ec, *Escherichia coli*; Mm, *Methanococcus maripaludis*; Tt, *Thermus thermophilus*; Hs, *Homo sapiens*. For EcMnmA, two possibilities are depicted (see text for details).

The eukaryotic enzyme Ncs6 (and its archaeal homolog NcsA) catalyzes sulfur transfer reactions at the C2 position of uridine at position 34 [52,53] (Figure 2A), and Ncs6 is thought to form a heterocomplex with the Ncs2 protein [54,55]. In some thermophilic bacteria and archaea, TtuA, a member of the same family of sulfurtransferases, catalyzes a similar reaction at a different tRNA position (position 54) [35,56]. Ncs6 (Ctu1 in humans) and TtuA are members of the ATP pyrophosphatase family that possess an ATP-binding pyrophosphate (PP) loop motif (Figure 3); hence, these enzymes are believed to catalyze an ATP-requiring two-step process (Figure 2A). Ncs6/TtuA utilizes an oxygen-sensitive Fe–S cluster in the catalytic center and a thiocarboxylate (R-COSH) as a sulfur donor, formed at the carboxy terminus of the specific sulfur carrier protein Urm1/TtuB. These sulfur carrier proteins also have roles as ancestral post-translational modifiers, similar to ubiquitin [57,58,59]. The C-terminus of Urm1/TtuB is adenylated by the E1-like enzyme Uba4/TtuC, then receives a persulfide from cysteine desulfurase via Tum1/TtuD and is subsequently thiocarboxylated. Through this process, Tum1/TtuD enhances the activity of cysteine desulfurase and directs the flow of sulfur toward s^2^U biosynthesis [60,61]. Recently, the molecular basis of Urm1 adenylation/thiocarboxylation by Uba4 was revealed by X-ray crystallography [62].

X-ray crystallography and EPR analyses clearly suggest that in the active center of TtuA, the [4Fe–4S] cluster is held by three conserved cysteines (Figure 3), leaving one iron atom free for ligand binding, which is important for the sulfur transfer reaction [56,63,64,65]. The C2 position of U54 is activated in the form of adenylate, and the thiocarboxyl group of TtuB is placed near the adenylation intermediate through binding to the iron–sulfur cluster [64], resulting in the transfer of the sulfur atom (Figure 2A(1)). Another mechanism has also been proposed in which sulfide ions released from TtuB-COSH bind to a vacant iron atom in the iron–sulfur cluster and are subsequently introduced into s^2^U (Figure 2A(2)). The latter pathway may also be used by organisms lacking the TtuB homolog, in which case intracellular free sulfide ions may be used. Data supporting the latter mechanism were provided by the state of the [4Fe–5S] cluster, which was determined by X-ray crystallography [56]. However, there is no direct proof that this sulfur atom is actually introduced into RNA. An in vivo study showed that TtuA recognizes a conserved sequence near U54 [66], but the structural basis of tRNA recognition by Ncs6/TtuA family enzymes has not yet been elucidated. The positively charged N- and C-terminus Zn finger domains (Figure 3) may contribute to tRNA binding [56,63,65].

It has also been reported that human Ncs6 (Ctu1) and the methanogenic archaeon enzyme NcsA bind the [3Fe–4S] cluster [67] (Figure 3). The cytidine C2 sulfurtransferase TtcA at position 32 of tRNA also belongs to the Ncs6/TtuA family subgroup and requires the [4Fe–4S] cluster for its activity [28,68] (Figure 3). Analysis of the differences in the reaction mechanisms of enzymes with different cluster types may lead to a more detailed understanding of the exact mechanisms of iron–sulfur cluster-dependent sulfurtransferases.

### 3.2. tRNA Uridine Sulfurtransferase MnmA

MnmA is an RNA sulfur-modifying enzyme that catalyzes sulfurization at C2 of uridine at position 34 in eubacteria [69]. In eukaryotic mitochondria, a homologous enzyme, Mtu1, is involved in s^2^U formation [70]. In *E. coli*, sulfur carrier proteins, such as TusA, the TusBCD complex, and TusE, are required for s^2^U formation [71]. TusA interacts with cysteine desulfurase IscS to receive sulfane sulfur and directs sulfur flow into the s^2^U biosynthetic pathway. The sulfane sulfur of TusA is then passed through TusD and TusE to the Cys199 residue in the active site of MnmA. However, many species do not require such intermediate persulfide carrier proteins and, in general, sulfur carrier proteins are not typically highly conserved between organisms [41]. MnmA possesses a PP loop motif and performs sulfurization in a two-step reaction via an adenylated intermediate (Figure 2B(1) and Figure 3). Nucleophilic attack of the adenylated intermediate by persulfide sulfur generates s^2^U, which is accompanied by the release of AMP [72]. X-ray crystallography analysis of the *E. coli* MnmA–tRNA complex has captured the adenylated intermediate involved in s^2^U formation [72], and the structure strongly supports the reaction mechanism described below. In a catalytic pocket isolated from bulk solvent, uridine reacts with ATP to form an adenylated intermediate, which reacts with terminal sulfur released from the persulfide of Cys199 with the help of another conserved cysteine (Cys102). In addition to the catalytic domain, the C-terminal domain recognizes the tRNA anticodon, whereas the central domain recognizes the minor groove formed by D and anticodon stems (Figure 3).

By contrast, MnmAs from eubacteria, such as thermophiles, photosynthetic bacteria, and pathogenic bacteria (e.g., *Mycobacterium tuberculosis),* were presumed to have iron–sulfur clusters and to be oxygen-sensitive, based on phylogenetic analysis, amino acid sequence features, and structure prediction [73] (Figure 3). This MnmA subtype has three conserved Cys residues in its catalytic center (CxxC—C type MnmA). Indeed, biochemical and electron paramagnetic resonance (EPR) spectroscopic analyses showed that MnmA from the thermophilic bacterium *Thermus thermophilus* is inactive under aerobic conditions but is active under anaerobic conditions, and it has a [4Fe–4S]-type cluster [73]. However, it remains unclear how the iron–sulfur cluster in *T. thermophilus* MnmA catalyzes the sulfur transfer reaction.

Intriguingly, it was reported that *E. coli* MnmA (DxxC—C type MnmA) can also bind an oxygen-sensitive [4Fe–4S] cluster via its Asp and two Cys residues in vitro, which is essential for s^2^U34 synthesis [74] (Figure 3). Contrary to the reaction mechanism proposed by an earlier study [72] (Figure 2B(1)), the authors proposed that the cluster serves to bind and activate hydrosulfide for nucleophilic attack on the adenylated nucleoside (Figure 2B(2)) as suggested for Ncs6/TtuA s^2^U-sulfurtransferases (see above). Although the activity of holo-EcMnmA in vitro is much higher than that of apo-EcMnmA, the activity seems to be somewhat low in this experimental condition [74]. While the activity may be enough to maintain the s^2^U34 level in vivo, some unidentified factors or conditions may be required for high specific activity. It will be interesting to investigate the possible differences in the reaction mechanisms of CxxC—C- and DxxC—C-type MnmAs.

### 3.3. tRNA Uridine Sulfurtransferase ThiI

The RNA sulfur-modifying enzyme ThiI, which is involved in s^4^U synthesis at position 8 of bacterial and archaeal tRNAs, also has a PP loop (Figure 3), and it is generally an iron–sulfur cluster-independent enzyme in many species [38,75,76,77,78]. ThiI uses ATP to activate the C4 atom of U as an adenylated intermediate (Figure 2C). In *E. coli*, the sulfur atom of the persulfide formed at the catalytic center of IscS is transferred to Cys456 of the rhodanese-like domain (RLD) of ThiI, and the sulfur atom is then incorporated into tRNA via assistance from a second catalytic Cys344 (Figure 2C(1) and Figure 3) [79,80,81,82]. The substrate tRNA is mainly recognized by the N-terminal ferredoxin-like domain (NFLD) and the thiouridine synthase, methylase, and pseudouridine synthase (THUMP) domain of ThiI (Figure 3) [83]. In addition to NFLD and THUMP domains, the catalytic domain with a Cys residue corresponding to Cys344 of EcThiI is conserved in ThiI from other species, while the RLD domain is conserved only in *E. coli* and a few closely related species [84]. Therefore, the catalytic mechanism of ThiI lacking the RLD domain remains to be determined. In addition, somewhat unusually, MmThiI of the methanogenic archaeon *Methanococcus maripaludis* possesses a CxxC motif and binds a [3Fe–4S] cluster that is essential for its activity (Figure 2C(2) and Figure 3) [67].

### 3.4. tRNA Adenosine Methylthiotransferase MiaB

2-Methylthio-A37 methylthiotransferases, such as bacterial MiaB and eukaryotic paralogs [85,86,87], belong to a subgroup of radical S-adenosyl-L-methionine (rSAM) enzymes with two iron–sulfur clusters [88]. These rSAM enzymes reductively cleave SAM to the 5′-deoxyadenosyl radical (5′-dA) and methionine, using a [4Fe–4S] cluster called the rSAM cluster (Figure 2D). The highly reactive 5′-dA radical then withdraws a hydrogen atom from the substrate to produce a substrate radical intermediate. The rSAM cluster and another [4Fe–4S] cluster, the auxiliary (Aux) cluster, are located close to each other, each tethered to the enzyme via three conserved cysteine residues [89,90]. Although the exact origin of the sulfur donor has not yet been determined, a reaction mechanism has been proposed in which a methyl group is transferred from another molecule of SAM to the apical sulfur atom of a (poly)sulfide bound to the Aux cluster, followed by substrate radical attack on the methylated sulfur atom to generate ms^2^A (Figure 2D) [91]. Another possible mechanism has recently been proposed in which the Aux [4Fe–4S] cluster is the direct sulfur source based on spectroscopic [92] and structural [93] studies. In this scheme, the cluster is decomposed for sulfur transfer to RNA and, therefore, requires restoration of the Aux cluster in MiaB. The newly discovered thioacetal structure of msms^2^A is formed by a second turnover involving hydrogen abstraction from the previously introduced methyl group of ms^2^A [23].

## 4. Degradation of Sulfur-Modified Bases

The demethylation pathways of RNA (and DNA) by dioxygenases, such as AlkB and TET, have been thoroughly investigated and their importance is widely accepted [94,95]. As for sulfur modification, the biosynthesis mechanism is well understood, as described above, but investigating the degradation of sulfur modifications is in its infancy. The stability of sulfur-modified bases against oxidative stress in vitro has been extensively analyzed, but the identification and characterization of intracellular dethiomethylation activity and the role of thiouracil desulfidase have only recently been reported (see below).

### 4.1. Stability of Sulfur-Modified Bases against Oxidative Stress

In vitro analysis has been performed on the chemical properties of s^2^U, specifically the desulfuration of s^2^U derivatives in monomeric form and within an oligo RNA/native tRNA [96,97,98]. Oxidation reactions with hydrogen peroxide and cytochrome C or Fe(II) results predominantly in 4-pyrimidinone nucleoside (h^2^U) rather than canonical U. Oxidation of s^2^U to h^2^U may affect codon–anticodon recognition because h^2^U has different hydrogen bonding properties to s^2^U, and h^2^U predominantly adopts a C2′-*endo* conformation in contrast to the preferred C3′-*endo* form of s^2^U. These studies are important for understanding the in vivo metabolism and functional changes of s^2^U derivatives.

### 4.2. Intracellular Dethiomethylation

It was recently reported that ms^2^C [29] and ms^4^U [30] are present in small amounts in cells. Since these modifications are increased by the addition of methylating reagents, such as methyl-methanesulfonate, these methylated thionucleosides may be alkylation-damaged bases resulting from nucleophilic s^2^C/s^4^U modification of tRNA. Sophisticated pulse-chase analysis of modified nucleoside dynamics, such as nucleic acid isotope labeling coupled to mass spectrometry (NAIL-MS), has revealed dethiomethylation activity in cells that yields canonical C or U, in addition to the enzymatic activity of AlkB, a demethylation enzyme with broad substrate specificity [95]. It is unclear whether dethiomethylation occurs spontaneously via nucleophilic attack by a water molecule or via an unidentified enzymatic activity. The authors proposed that spontaneous dethiomethylation of ms^2^C is the most plausible scenario, considering the electrophilic nature of C2 in ms^2^C.

### 4.3. Thiouracil Desulfidase TudS

The iron–sulfur cluster protein TudS, possessing thiouracil desulfidase activity, was recently discovered and elegantly characterized using structural and biochemical approaches [99]. TudS catalyzes desulfuration of 4-thiouracil or 2-thiouracil to form canonical uracil. TudS possesses a [4Fe–4S] cluster bound by only three cysteine residues of the catalytic center. Incubation of TudS crystals with the substrate 4-thiouracil led to trapping of a catalytic intermediate, a [4Fe–5S]-type cluster, with a sulfur atom bound to the fourth iron atom of the [4Fe–4S] cluster. The authors proposed a mechanistic scheme for the desulfuration reaction of thiouracil in which a [4Fe–4S] cluster binds and activates the sulfur atom of the substrate nucleobase (Figure 2E). The biological significance of TudS family enzymes has not been established, but they are proposed to function in a salvage pathway for uracil biosynthesis, since TudS was identified through in vivo screening [100]. The TudS gene (formerly DUF523) was screened from a metagenomic library, and the gene product allowed for the conversion of 2-thiouracil to uracil in a uracil auxotroph strain of *E. coli*. This in vivo degradation activity of a sulfur-containing nucleobases is an important discovery, and TudS may share a similar reaction mechanism with cysteine desulfidase, a [4Fe–4S] cluster-containing enzyme that decomposes L-Cys to hydrogen sulfide, ammonia, and pyruvate [101]. The presence of a common intermediate (a [4Fe–5S]-type cluster) in enzymes related to both biosynthesis (TtuA/Ncs6 and MiaB families) and biodegradation (TudS) is quite interesting and is an intriguing property of [4Fe–4S] clusters, although in the case of biosynthesis enzymes, it should be clarified whether the sulfur atom is actually incorporated into the substrate.

## 5. Sulfur Modification Abnormalities and Disease

**Figure 4:**
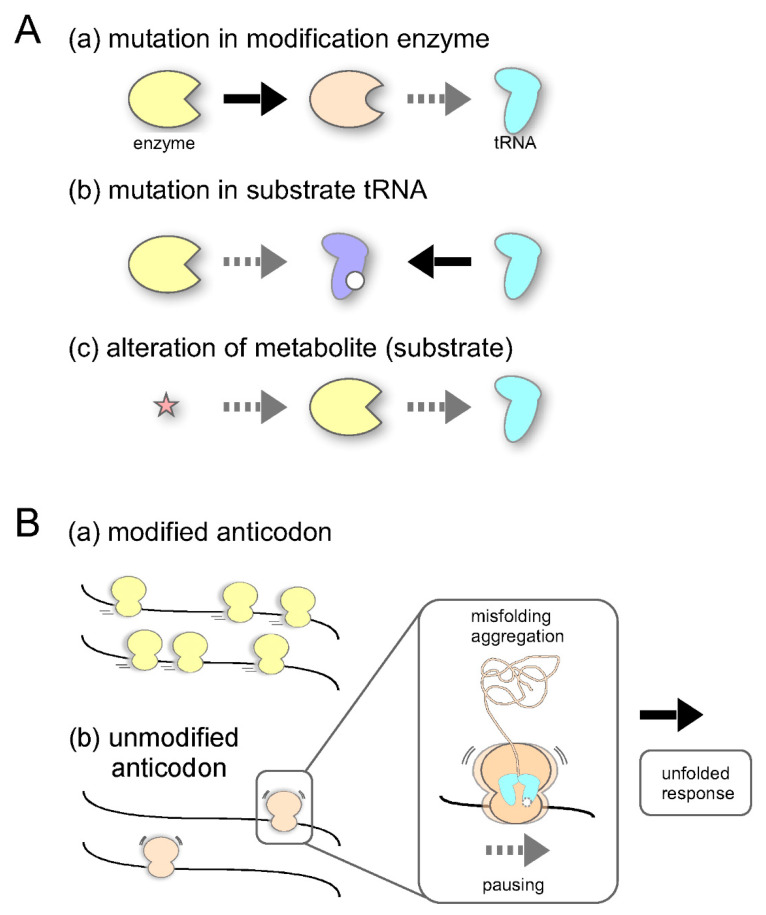
Disorders in RNA modification: (**A**) Three pathways via which aberrations can occur in tRNA modification. (**B**) (a) mRNAs that have many more XAA codons (for Lys, Glu, and Gln) can be effectively read with modified anticodons, which leads to proper folding of the proteome. (b) Only the translation of codon-biased genes is affected if there is no modification in the anticodon. Slow translation causes misfolding and aggregation of the proteome, which leads to the unfolded response.

Disorder of RNA modification can lead to diseases in three main ways (Figure 4A): (a) mutations in genes of RNA modification enzymes, (b) mutations in substrate RNAs, and (c) alteration of metabolites affecting the biosynthesis of enzyme co-substrates and cofactors. Codon-specific translational regulation with specific mRNAs through (sulfur-)modification of tRNAs has been confirmed in a model system, and its relevance to human diseases has been confirmed in several cases. For a comprehensive overview of diseases associated with RNA modification, there are excellent reviews available [102,103]. Examples of recent advances related to sulfur modification are described below.

### 5.1. Cancer

Accumulation of ribosomes at AAA and CAA codons in mRNAs occurs in yeast lacking the 5-methoxycarbonylmethyl (5-mcm) and/or 2-thio modification of mcm^5^s^2^U34, and ribosome accumulation at AAA, CAA, and GAA codons in nematode worm mutants lacking the 2-thio modification [20,104]. Pausing of the ribosome causes protein misfolding and aggregate formation, and overexpression of unmodified tRNAs reverses these defects [20], showing that an optimal speed for translation supported by tRNA wobble modifications is very important for maintaining proteome integrity (Figure 4B).

It has been shown that codon-specific regulation of translation promotes resistance to targeted therapy in human cancer [5]. BRAF^V600E^-expressing melanoma cells are dependent on U34 modification enzymes for survival (Elp3 and Ctu1 for 5′-modification and 2-thiolation, respectively). Activation of the PI3K signaling pathway, a major mechanism of acquired resistance against agents targeting MAPK, increases the expression of U34 modification enzymes. Translation of HIF1A mRNA, which is codon biased, is promoted by these anticodon modifications, and a metabolic transition to glycolysis occurs via up-regulation of HIF1α. This study showed that RNA modification enzymes promote the survival and resistance to therapy of cancer cells by specific modulation of mRNA translation.

### 5.2. Diabetes

CDKAL1, a homolog of bacterial MiaB [105], is a 2-methylthio transferase responsible for the synthesis of 2-methylthio-N6-threonylcarbamoyladenosine (ms^2^t^6^A37) in cytosolic tRNA^Lys^ in eukaryotes [6,87]. Type 2 diabetes (T2D) is the most frequent type of diabetes, and genetic variations in the CDKAL1 gene are associated with T2D [106]. Defects in insulin production and insulin resistance cause high levels of blood sugar, a characteristic feature of T2D. CDKAL1 knockout mice display pancreatic islet hypertrophy, decreased insulin secretion, and impaired blood glucose control, which are major T2D-associated phenotypes [6]. Misreading of a critical Lys codon in proinsulin seems to result in a reduction in glucose-stimulated proinsulin synthesis. It was recently reported that iron regulatory protein 2 (Irp2) regulates insulin production through iron (sulfur)-mediated CDKAL1-catalyzed tRNA modification [107]. This study revealed an important link between iron deficiency and insulin processing. Irp2 regulates transferrin receptor 1 (an iron uptake protein) and ferritin (an iron storage protein). Therefore, Irp2 loss results in functional iron deficiency in β cells. This impairs iron–sulfur cluster biosynthesis and reduces the activity of the iron–sulfur protein CDKAL1, eventually resulting in a reduction in insulin synthesis. Therefore, mice lacking Irp2 develop diabetes.

### 5.3. Mitochondrial Disease

Mitochondrial disease is a symptom of mitochondrial dysfunction that mainly affects the functions of brain and skeletal muscle, where energy demands are relatively high [108]. Mitochondrial myopathy, encephalopathy, lactic acidosis, and stroke-like episodes (MELAS) and myoclonus epilepsy associated with ragged-red fibers (MEERFs) are mitochondrial diseases caused by point mutations in the mt-tRNA^Leu(UUR)^ and mt-tRNA^Lys^ genes, respectively. Because point mutations in tRNAs may affect the recognition of tRNAs by U34 modifying enzymes, mt-tRNAs lack the normal 5-taurinomethyl-(2-thio)uridine (τm^5^(s^2^)U) modification at position 34, which results in defects in mitochondrial translation [109,110] and leads to disease progression [111]. Ribosome profiling in MELAS cells showed that ribosomes tend to accumulate at the Leu UUG codon, and loss of taurine modification reduces the translational capacity of UUG codons [112].

The importance of ms^2^i^6^A37 in mt-tRNAs for mitochondrial translation has also been revealed [7]. CDK5RAP1 is a eukaryotic 2-methylthio transferase responsible for the synthesis of 2-methylthio-N6-isopentenyladenosine (ms^2^i^6^A37) in mitochondrial tRNA-Trp, -Phe, -Tyr, and -Ser. Methylthio modification in mt-tRNAs was absent and mitochondrial translation was lower in MEF cells and tissues from CDK5RAP1 knockout mice. Deficiency of the methylthio modification accelerates myopathy and cardiac dysfunction. Methylthio modification levels were also lower in blood cells from MELAS patients, and because MELAS mutant cells exhibited elevated oxidative stress [113], the enzymatic activity of CDK5RAP1 may be affected via disruption of its oxidation-liable iron–sulfur clusters in the catalytic center [87,105].

## 6. Related Pathways and Evolutionary Implications for Sulfur Metabolism

Identification of the components and reaction mechanisms of sulfur metabolism related to sulfur nucleosides has revealed their common characteristics and remarkable diversity. Several families of sulfur carrier proteins particular to each pathway and species have been identified. Many kinds of activated sulfur species are now known, such as protein-bound persulfide and thiocarboxylate, and hydrosulfide bound to an iron atom of iron–sulfur clusters.

An increasing number of sulfurtransferases related to PP loop-containing RNA sulfurtransferase are involved in the biosynthesis of cofactors and secondary metabolites [1]. For example, in the biosynthesis of a so-called nickel pincer nucleotide cofactor, the LarE protein catalyzes sulfur insertion into the nucleotide precursor (i.e., transformation of carboxylate to thiocarboxylate) [114]. The lactate racemase LarA from *Lactobacillus plantarum* possesses a sulfur-containing cofactor, pyridinium 3-thioamide-5-thiocarboxylic acid mononucleotide with nickel bound to the C4 carbon atom of the pyridinium ring and the two sulfur atoms of the cofactor, and the His200 sidechain of LarA [115]. LarE, a member of the PP-loop pyrophosphatase family containing a SGGxDS motif, is distantly related to RNA sulfurtransferases such as ThiI, MnmA, and Ncs6/TtuA/TtcA [116]. *L. plantarum* LarE has only one Cys residue in its catalytic center, and the sulfur atom of this Cys residue is used as the sulfur donor; thus, LpLarE is a ‘suicide enzyme’. Most other LarE homologs have CxxC—C motifs that are thought to be required for iron–sulfur cluster or persulfide binding and may be multiple turnover enzymes [117], although experimental validation is required.

Recently, the biodegradation pathway of thiolated nucleosides and its enzymes have been characterized. Intriguingly, the storage and recycling system for sulfur atoms in plants has recently been reported [118]. It is now known that *Arabidopsis thaliana* uses a retrograde pathway of sulfur atoms from secondary metabolites (glucosinolates, compounds with more than two sulfur atoms) to primary metabolites (cysteine) and that utilizing a secondary metabolite as a storage of sulfur atom is physiologically advantageous under sulfur-poor conditions. Coupled regulation of biosynthesis and degradation may also be important for cellular sulfur-metabolism including RNA sulfur-nucleosides.

The primitive Earth had a reductive environment with minimal oxygen, but it now has an oxidative environment. However, life did not abandon the use of oxygen-sensitive iron–sulfur clusters that appear to be disadvantageous for the activity of several enzymes including RNA sulfurtransferases. In addition to their catalytic functions, iron–sulfur clusters may serve as sensors of oxidative stress, and damaged iron–sulfur clusters are thought to be actively repaired in cells [119]. Further, more in-depth investigations into the mechanisms of sulfur modifications in tRNAs may increase understanding of the evolution of life on Earth, which has evolved in response to the environmental changes that occurred after molecular oxygen accumulated with the advent of photosynthesis. Coevolution with iron–sulfur cluster repair mechanisms may have been crucial.

## 7. Concluding Remarks and Perspectives

Remarkable progress has been made in research on the biosynthesis and degradation mechanisms of sulfur modification of tRNAs, which has provided insight into the functions of sulfur modification of tRNAs in translation. However, except in a few cases, the direct relationship between translational defects and onset/progression of certain diseases remains to be investigated. One of the key aspects of sulfur modifications themselves, and their related biosynthesis pathways involving unstable activated intermediates, such as persulfide and thiocarboxylate and enzymes with metal cofactors, is the characteristic nature of the susceptibility to oxidative stress. Determination of the spatiotemporal modification status of individual tRNAs may be a basis for understanding the onset and progression of diseases related to sulfur modification of RNAs. For this, improved high-throughput methods will be needed to precisely determine the intact status of sulfur modification at each position of tRNAs and the abundance of tRNAs. In addition, in order to understand homeostasis and the regulation of sulfur modifications in RNAs within the global network of sulfur metabolites in the cell, identification of predicted sulfur carrier proteins mediating the biosynthesis of sulfur modifications will be needed, and sulfur flow in the cell will need to be quantified.
